# Hybrid Pulmonary Rehabilitation Improves Cardiorespiratory Exercise Fitness in Formerly Hospitalised Long COVID Patients

**DOI:** 10.3390/jcm14124225

**Published:** 2025-06-13

**Authors:** Nikolaos Chynkiamis, Angelos Vontetsianos, Christina Anagnostopoulou, Christiana Lekka, Maria Ioanna Gounaridi, Evangelos Oikonomou, Manolis Vavuranakis, Nikoleta Rovina, Petros Bakakos, Nikolaos Koulouris, Georgios Kaltsakas, Ioannis Vogiatzis

**Affiliations:** 1 Rehabilitation Unit, 1st University Department of Respiratory Medicine, “Sotiria” Hospital, Medical School, National and Kapodistrian University of Athens, 11527 Athens, Greece; agelvonte@gmail.com (A.V.);; 2 Thorax Research Foundation, 11521 Athens, Greece; 3 3rd Department of Cardiology, “Sotiria” Hospital, Medical School, National and Kapodistrian University of Athens, 11527 Athens, Greece; 4 Lane Fox Respiratory Service, Guy’s and St Thomas’ NHS Foundation Trust, London SE1 9RT, UK; 5 Centre of Human and Applied Physiological Sciences, Faculty of Life Sciences and Medicine, King’s College London, London WC2R 2LS, UK; 6 Department of Sport, Exercise and Rehabilitation, Faculty of Health and Life Sciences, Northumbria University Newcastle, Newcastle upon Tyne NE1 8ST, UK

**Keywords:** COVID-19 survivors, pulmonary rehabilitation, cardiorespiratory responses

## Abstract

**Background/Objectives:** Supervised pulmonary rehabilitation (PR) is effective in improving cardiorespiratory fitness in non-hospitalised individuals with long COVID. However, there is limited evidence regarding PR-induced improvements in cardiorespiratory parameters in previously hospitalised COVID-19 survivors. This study aimed to investigate the effect of a hybrid PR programme (outpatient followed by a digital intervention) on exercise tolerance, cardiorespiratory adaptations, functional capacity and quality of life outcomes in previously hospitalised COVID-19 survivors. **Methods:** Forty-two patients (age (mean ± SD): 57 ± 12 yrs) with excessive fatigue due to long COVID (FACIT score (26 ± 10) were allocated to PR (n = 27) or usual care (UC) (n = 15) 140 ± 75 days from hospital discharge. PR consisted of 8 outpatient sessions (twice weekly for 4 weeks) followed by 24 home-based sessions (3 times/week for 8 weeks). Patients in the UC group were instructed to be physically active. Exercise tolerance was assessed by cardiopulmonary cycling testing to the limit of tolerance. **Results:** Following the completion of the hybrid PR programme, peak work rate (WRpeak) and peak oxygen uptake (VO_2_peak) were, respectively, improved in the PR group by 19 ± 10 Watt (*p* = 0.001) and by 2.4 ± 3.0 mL/kg/min (*p* = 0.001). Furthermore, in the PR group, the 6 min walk distance was increased by 72 ± 69 metres (*p* = 0.001). FACIT and mMRC scores were also improved in the PR group by 15 ± 10 (*p* = 0.001) and by 1.4 ± 1.0 (*p* = 0.001), respectively. In the UC group, only the mMRC score was improved by 0.7 ± 1.0 (*p* = 0.008). **Conclusions:** The application of a hybrid PR programme was beneficial in improving cardiorespiratory exercise fitness, functional capacity and quality of life in previously hospitalised COVID-19 survivors.

## 1. Introduction

As of February 2025, more than 700 million people have been affected by SARS-CoV-2 globally [1]. Since January 2020, Greece has reported over 5.5 million confirmed COVID-19 cases and more than 35,000 deaths occurring within the first 28 days of infection [2]. Accordingly, it is apparent that the majority of patients fully recover, even those requiring hospital admission; however, a proportion of patients present with persistent symptoms at least three months after the acute illness, a condition defined by NICE as long COVID [3]. The most prevalent symptoms include long-standing exertional fatigue, dyspnoea, muscle and joint pain, sleep disturbances, short-term memory loss and brain fog [4].

Long COVID affects a large group of patients, and according to the WHO, it imposes a great burden on healthcare systems worldwide [1]. Several studies have highlighted the adverse effects of long COVID on exercise tolerance [5,6,7], functional capacity and cardiorespiratory fitness in hospitalised COVID-19 survivors [8]. Indeed, in these survivors, cardiopulmonary exercise testing (CPET) has shown significant cardiorespiratory impairment lasting for several months post-hospital discharge [6,9,10,11]. This finding alone highlights the potential benefit of exercise-based PR in the recovery of cardiorespiratory fitness. Accordingly, international and national guidelines have been issued recommending the implementation of pulmonary rehabilitation (PR) programmes for the management of patients with long COVID [12,13] including both outpatient and tele-rehabilitation modalities in adult survivors of COVID-19 [14].

Recently published studies have reported that both hospital-based and digital PR programmes improve quality of life, functional capacity and exercise tolerance assessed by field walking tests in patients with long COVID [5,8,15,16,17,18,19]. More recent work provides evidence of positive effects of outpatient supervised PR programmes on cardiorespiratory exercise fitness, albeit in non-hospitalised COVID-19 survivors [20,21,22,23,24]. Therefore, it remains inconclusive whether PR could induce significant cardiorespiratory adaptations during exercise in previously hospitalised COVID-19 survivors who present with persistent symptoms.

Furthermore, long COVID patients are relatively young and economically active, and thus, it is not always easy for them to attend supervised PR programmes lasting more than few weeks. Thus, it is important to educate long COVID patients to self-manage their symptoms [25,26] by applying hybrid PR programmes that could potentially combine an initial phase of outpatient PR followed by a home-based digital intervention.

The aim of the present study was to investigate the effect of a hybrid PR programme (including outpatient and home-based sessions) on cardiorespiratory exercise fitness, functional capacity and quality of life outcomes in previously hospitalised patients with long COVID. It was hypothesised that the application of a hybrid PR programme would improve cardiorespiratory fitness alongside exercise tolerance and symptom intensity.

## 2. Materials and Methods

### 2.1. Study Design

**Figure 1:**
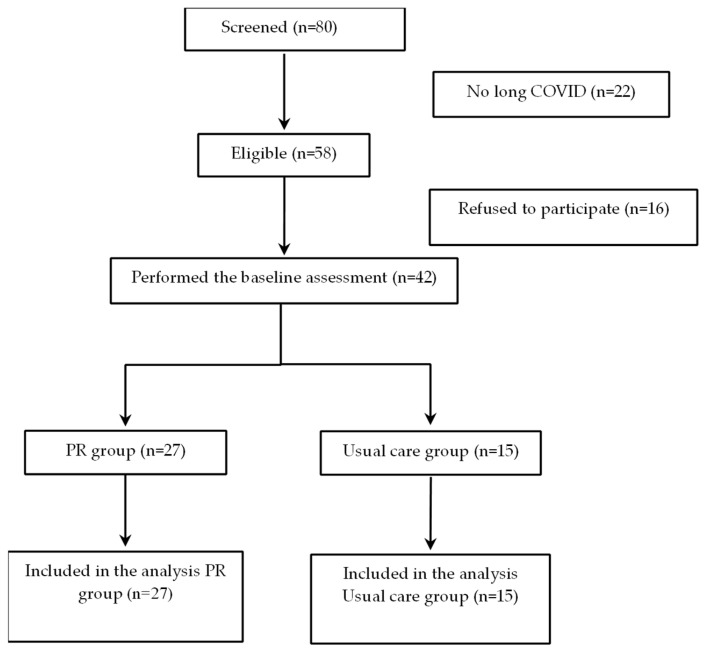
Study flow chart.

This feasibility, single-centre, propensity-matched study assessed the effect of a hybrid PR programme (consisting of 8 outpatient and 24 home-based PR sessions) on exercise tolerance, cardiorespiratory fitness, functional capacity and quality of life in previously hospitalised patients with long COVID. The study took place from August 2022 to June 2023 at the 1st University Department of Respiratory Medicine of Athens Medical School in Greece. All patients included in the study were hospitalised due to confirmed SARS-CoV-2 infection, as verified by PCR testing upon admission to the emergency department. Hospitalisation was warranted due to acute respiratory failure, not merely a positive test. These cases were managed in accordance with national and international clinical guidelines [27]. We did not include patients diagnosed solely via rapid antigen tests or self-administered testing. The diagnosis of COVID-19 in this cohort was made in a clinical context, where PCR testing was used as part of routine hospital admission protocols and aligned with national diagnostic guidelines at the time. The diagnosis of long COVID was made in accordance with established criteria [28], specifically the persistence of fatigue for at least three months following hospital discharge. This diagnosis was determined by a respiratory specialist during structured follow-up assessments conducted at one, three, six, and twelve months post discharge. Exclusion criteria included major cardiovascular or neurologic events, uncontrolled congestive heart failure, a history of psychiatric illness without sufficient medical supervision and a history of active cancer. PR was offered to all patients who met the inclusion criteria. Those who were unable to attend the supervised sessions due to transport constrains or work commitments were offered to participate in the usual care group including assessments at baseline and 12 weeks afterwards (Figure 1). Patients’ assessments were performed prior and following the completion of the 12-week hybrid PR programme and included comprehensive respiratory function testing, assessment of cardiorespiratory fitness via a cardiopulmonary exercise test (CPET), respiratory and peripheral muscle strength and evaluation of functional capacity, quality of life and daily physical activity.

### 2.2. Respiratory Function Assessment

Respiratory function testing, including spirometry as well as measurement of static lung volumes using the multi-breath nitrogen washout technique and diffusion capacity for carbon monoxide using the single-breath nitrogen washout technique, was performed with a metabolic cart (Vmax Encore 22: Sensor Medics, Yorba Linda, CA, USA). Predicted values for the aforementioned measurements were in accordance with the ERS guidelines [29]. A plastic semi-rigid flanged mouthpiece fitted to a metallic stem incorporating a 3-way tap, which was manufactured according to the design of Ringqvist [30,31], was used to determine the maximum static inspiratory (PImax) and expiratory (PEmax) mouth pressures.

### 2.3. Body Mass Composition, Peripheral Muscle Strength and Quality of Life

Body mass composition was evaluated via the bio-impedance method (Bodystat 1500, Bodystat LTD, Isle of Man, UK). Handgrip strength was measured using a handgrip dynamometer (KERN & Sohn GmbH, Balingen, Germany), and quadriceps muscle force (QF) was assessed using a strain gauge myometer device (MIE, Medical Research LTD, Leeds, UK). FACIT, CAT, HADS, SF-36 and the Revised Impact of Events Scale questionnaires were used to assess quality of life, symptom intensity and levels of anxiety and depression.

### 2.4. Incremental Cycle Ergometer Exercise Test

Patients performed a ramp incremental exercise protocol to the limit of tolerance (CPET) on a cycle ergometer (Vyaire Medical GmbH, Hoechberg, Germany). During the first 3 min, resting measurements were obtained. Following the resting phase, a 3 min warmup phase of unloaded pedalling was performed, and subsequently, work rate was increased by 10–25 Watts per minute up to the limit of tolerance. Oxygen saturation (SpO_2_%) was recorded throughout the CPET using an ear pulse oximeter connected to the metabolic cart (Vyntus ONE, Vyaire Medical GmbH, Hoechberg, Germany). Blood pressure was recorded at predefined time points via an automatic cuff connected to the metabolic cart. Finally, perceived dyspnoea and leg discomfort were reported every 3 min using the modified Borg 1–10 scale [32]. Iso-work rate was defined as the workload corresponding to the point of exercise termination during the shorter of the two incremental cycle tests (i.e., pre- or post-intervention), allowing for comparison of physiological responses at the same absolute intensity across both tests.

### 2.5. Functional Capacity

Participants were asked to complete the 6 min walk test (6MWT) on a 20-metre hospital corridor according to the American Thoracic Society guidelines [33]. SpO_2_%, heart rate (HR), dyspnoea and leg discomfort were evaluated at rest and after the completion of the 6MWT. The Short Physical Performance Battery Test (SPPB) and the 60 s sit-to-stand test were also performed to provide additional data regarding functional capacity.

### 2.6. Daily Physical Activity

All patients were instructed to install in their smartphones a free application (Step counter, Leap Health Fitness Limited, Hong Kong) [34] in order to record their daily physical activity levels for seven consecutive days, one week before the beginning of the programme and one week following its completion. They were encouraged to have their phone in their pocket as long as possible during this period in order to avoid data loss.

### 2.7. Exercise-Based PR Protocol

During the outpatient PR programme, all participants underwent 8 supervised exercise training sessions consisting of 30 min of interval aerobic exercise on electromagnetically braked cycle ergometers (CateyeErgociser, ECI600; Osaka, Japan). Initial exercise intensity was equivalent to 100% of the baseline peak work rate (WRpeak) achieved by the participants during the baseline CPET. Following the aerobic training, resistance exercises for the upper and lower limbs were performed. During each training session, dyspnoea and leg discomfort were recorded using the modified Borg scale [32], whereas HR and SpO_2_% were continuously monitored by a portable pulse oximeter (Beurer PO 35, Beurer GmbH, Ulm, Germany). Based on symptom intensity reported by the participants at the end of each exercise session, cycling intensity was progressively increased by 5–10% of the WRpeak recorded at baseline. When dyspnoea and leg discomfort were scored ≥4 on the Borg scale, the exercise intensity remained the same. The remote 24 home-based PR sessions included 30 min walking with an individualised target of steps (recorded via the mobile app installed onto the patients’ mobile phones). Step count, leg discomfort and dyspnoea symptoms were recorded by the patient daily using a home activity diary. The researchers set new step targets based on the steps completed and the symptoms reported by the patients. When dyspnoea and leg discomfort were both <4, the weekly target of steps was increased by 5–10% of the baseline step count; otherwise, the target remained the same.

### 2.8. Statistical Analysis

Using the mean difference in the improvement in peak work rate between the intervention and control group (42 Watt), the standard deviation (SD) (50 Watt), an alpha significance level of 0.05 (2-sided), 80% power and a group allocation ratio 0.5, a minimum total sample size of 54 patients (36 in the intervention group and 18 in the control group) would be sufficient to detect significant differences in peak work rate.

Data are expressed as the mean ± SD unless otherwise stated. Normal distribution of the data was checked with the Shapiro–Wilk test. The independent *t*-test was used in order to make comparisons of baseline characteristics. The effect of the hybrid PR programme (i.e., pre- to post-rehabilitation) and the differences between the two groups were detected using two-way ANOVA with repeated measures. The LSD post hoc correction method was used where appropriate. Dependent sample *t*-tests were performed to compare the responses at iso-work within each group. A *p*-value < 0.05 was considered significant. Statistical analysis was performed using IBM SPSS 22 statistical software.

## 3. Results

Twenty-seven and fifteen patients were included in the PR and UC groups, respectively. The demographic characteristics of the participants are presented in Table 1. There were not any significant differences in baseline characteristics between the intervention and UC groups at the outset of the study.

WRpeak and VO_2_peak were, respectively, improved in the PR group by 20% (*p* = 0.001) and 15% (*p* = 0.001) following the completion of the PR programme (Table 2). In contrast, there was no difference in WRpeak (*p* = 0.205) and VO_2_peak (*p* = 0.565) in the usual care group between baseline and the three-month reassessment (Table 2). Following completion of the PR programme, fatigue, assessed via the 1–10 Borg scale at peak exercise, was lower by 1.1 ± 2.2 units compared to baseline. This is an important finding considering that peak work capacity following the completion of the hybrid PR programme was increased by approximately 20 watts (Table 2).

At iso-work during the incremental cycling test prior to PR at baseline, patients in the PR group demonstrated lower levels of minute ventilation (by 6.8 ± 2.1 l/min, *p* = 0.003) secondary to a lower breathing frequency (by 6 ± 2 breaths/min, *p* = 0.001) (Table 3). As a result, at iso-work, compared to baseline peak work, inspiratory time (Ti), expiratory time (Te) and duty cycle time (Ttot) were greater in the PR group (by 0.11 ± 0.04 s (*p* = 0.013) by 0.15 ± 0.05 s (*p* = 0.007) and 0.26 ± 0.09 (*p* = 0.007), respectively (Table 3). In the PR group, compared to baseline peak work, dyspnoea and leg fatigue were lower at post-discharge iso-work by 1.5 ± 1.3 (*p* = 0.001) and 2.4 ± 2.2 (*p* = 0.001), respectively (Table 3). There were no differences in the UC group in any of the parameters included in the analysis (Table 3).

**Figure 2:**
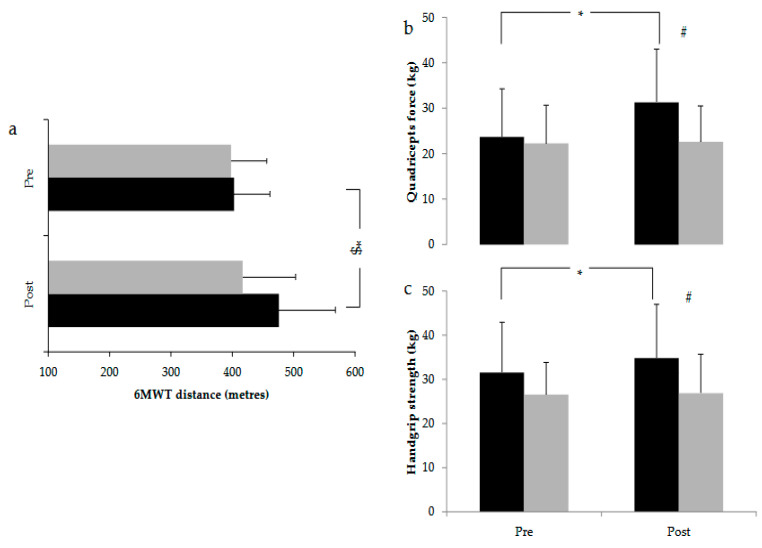
The effect of the application of the hybrid PR programme on (**a**) the distance covered during the 6MWT, (**b**) quadriceps force and (**c**) handgrip strength in the PR group (black bars) and the usual care group (grey bars). * denotes a statistically significant difference within the same group. # denotes a statistically significant difference between the PR and usual care groups at the same time point. $ denotes a clinically important difference within the same group.

Following PR, the 6 min walk distance was increased by 72 ± 69 metres (*p* = 0.001) (Figure 2a) in the PR group, exceeding the minimum clinical important difference of 26 or 34 metres that is reported for patients with chronic respiratory disease [35,36]. There was no difference in the distance covered during the 6 min walk test in the control group after the 12-week follow-up (*p* = 0.274) (Figure 2a).

Quadriceps force (QF) and handgrip force were increased in the PR group following the completion of the PR program by 4.0 ± 3.8 kg (*p* = 0.001) and 3.3 ± 3.1 kg (*p* = 0.001) (Figure 2b). In contrast, there was no difference in QF (*p* = 0.726) and handgrip strength (*p* = 0.815) in the control group between baseline and 12-week reassessment (Figure 2c). Regarding respiratory muscle strength, PImax and PEmax values were similar between the groups before and after the completion of the PR programme.

**Figure 3:**
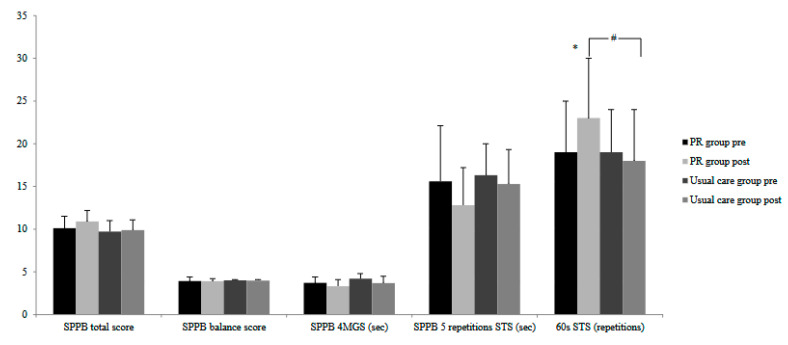
Short Physical Performance Battery test (SPPB) total score, its components (balance score, 4 m walk test and 5-repetition sit-to-stand test) and repetitions performed during the 60 s sit-to-stand test in the PR and usual care groups prior to and following the completion of the PR programme. * denotes a statistically significant difference within the same group. # denotes a statistically significant difference between the PR and usual care groups at the same time point.

Compared to baseline, there was a trend of improvement in the SPPB total score in the PR group (ANOVA *p* = 0.065); however, none of the SPPB individual components were significantly affected by the hybrid PR programme (Figure 3). Finally, the number of repetitions during the 60 s sit-to-stand test was significantly increased in the PR group by 4.4 ± 3.3 repetitions (*p* = 0.001) (Figure 3). There was no difference in the number of repetitions during the 60 s sit-to-stand test in the control group (*p* = 0.814).

Quality of life and daily physical activity data are presented in Table 4. General fatigue score, assessed via the Functional Assessment of Chronic Illness Therapy (FACIT) questionnaire, and symptom intensity score, assessed via the CAT questionnaire, were improved in the PR group by 15 ± 10 (*p* = 0.001) and 7 ± 6 (*p* = 0.001), respectively (Table 4). Additionally, chronic dyspnoea assessed via the modified Medical Research Council Scale (mMRC) was improved in the PR group by 1.4 ± 1.0 (*p* = 0.001) (Table 4). In contrast, in the control group, only the mMRC score was improved at the 12-week assessment compared to baseline by 0.7 ± 1.0 (*p* = 0.008) (Table 4).

Transport constraints and work obligations were the main factors which prevented patients from attending all of the supervised hospital-based PR sessions. On the contrary, the adherence rate during the remote exercise sessions was very high as patients completed 23 out of 24 sessions (94%).

## 4. Discussion

The novel finding of the present study is that a hybrid, exercise-based PR programme, comprising 4 weeks of supervised hospital-based exercise sessions followed by 8 weeks of home-based self-managed walking sessions, was effective in improving exercise tolerance, cardiorespiratory fitness and quality of life parameters in previously hospitalised COVID-19 survivors with long COVID.

Long COVID survivors in our cohort had an impaired VO_2_ peak at baseline corresponding to approximately 65% of the predicted normal level, and this was well observed in this population post hospital discharge [6,9,10,11]. However, 12 weeks of exercise combining outpatient and home-based PR sessions significantly increased VO_2_ peak, peak work rate and peak minute ventilation. This is a novel finding highlighting the value of exercise-based PR in previously hospitalised COVID-19 survivors. Despite the improvement in VO_2_ peak by 15% compared to the baseline value, VO_2_ peak was still suboptimal at the end of the PR programme, thereby highlighting the potential need for lengthier PR programmes. Significant cardiorespiratory adaptations were accompanied by clinically meaningful reductions in scores for dyspnoea and fatigue at peak exercise, indicating improved ventilatory and metabolic efficiency. In support of this notion, lower ventilatory demand was observed at iso-work rate during the incremental CPET.

Few studies have assessed the effect of PR on cardiorespiratory fitness [20,21,22,23,24,37,38,39,40]. The results of these studies were not consistent in terms of showing a significant improvement in peak oxygen uptake following PR [41]. Specifically, three RCTs investigating the effects of PR on cardiorespiratory fitness failed to demonstrate improvements in VO_2_ peak following the completion of the programme [23,24,39], whilst one RCT demonstrated an improvement of 2.7 mL/min/kg [21]. In contrast, five non-RCTs reported a mean increase in VO_2_ peak of 2.9 mL/min/kg [20,22,37,38,40]. The latter is in line with the findings from our cohort as we report an increase in VO_2_ peak by 2.4 mL/min/kg, even though our study participants performed only eight outpatient PR sessions. We speculate that the high-intensity interval exercise performed during the 4-week outpatient PR programme in our cohort was sufficient to similarly improve aerobic and work capacity compared to the studies where moderate-intensity PR was applied for 12 weeks [20,21,22,37,38,40]. Interestingly, patients in the PR group exhibited lower scores for fatigue at peak exercise at the end of the PR programme compared to baseline, indicating more efficient oxygen uptake and utilisation.

Previous studies reported improvements in walking distance during the 6MWT following the completion of a PR programme [21,22,42,43,44,45]. More specifically, numerous studies reported that the average distance covered during the 6MWT increased by 62 m [21,22,42,43,44,45]. Our findings are in accordance with the current literature, demonstrating an improvement of 72 metres in the distance covered during the 6MWT in the intervention group.

According to the existing literature, peripheral muscle strength including both handgrip strength and quadriceps force are decreased in patients with long COVID. However, the application of either outpatient or tele-rehabilitation PR sessions restores muscle strength to normal levels [23,25,46]. In our cohort, patients in the PR group demonstrated a similar increase to that reported in the literature regarding handgrip strength [25,46]. In contrast, quadriceps strength showed similar improvements compared to the study by Gloeckl and colleagues [25], whilst it was greater compared to the results reported by Jimeno-Almazan and colleagues [23] following the completion of the hybrid PR programme. The discrepancy between our findings and the findings from the study by Jimeno-Almazan and colleagues [23] can be explained by the fact that the main intervention applied in their study was respiratory muscle training. In contrast, the improvement reported in the present study in both handgrip and quadriceps strength could be explained by the efficient combination of aerobic and resistance training sessions performed in the hospital as well as at home.

Both outpatient [47] and tele-rehabilitation [48] programmes improve functional capacity evaluated by the 30 s sit-to-stand test and SPPB scores in patients with long COVID. In a particular study, repetitions during the 30 s sit-to-stand test were increased by seven following a 6-week home-based low-intensity PR programme [49], whilst SPPB total score and its components were improved following an 8-week rehabilitation programme [50]. Similar patterns of improvement were demonstrated in our cohort as 60 s sit-to-stand performance was increased by four repetitions and SPPB total score was increased secondary to better performance in the 4-metre gait test and during the five sit-to-stand repetitions.

The beneficial effects of pulmonary rehabilitation programmes on the quality of life (QoL) and emotional status of long COVID patients are well established [46,51,52,53,54]. In line with the current literature, our patients demonstrated improved scores in FACIT, CAT, and mMRC questionnaires, confirming the positive effects of PR programmes on these aspects. Moreover, the hybrid nature of our PR programme resulted in improvements in patients’ symptom intensity, particularly fatigue and dyspnoea. Regarding emotional status, long COVID patients in the PR and usual care groups reported similar reductions in depression and anxiety levels at the 12-week follow-up, but did not reach statistical significance.

An important advantage of the hybrid nature of our PR programme was the high adherence rate observed in our cohort during the home-based sessions. As mentioned earlier, patients were generally young and economically active, which would have prevented them from attending an outpatient programme, especially for a long period of time. The high adherence rate observed during the remote sessions is in accordance with other studies where the effects of home-based rehabilitation programmes were proven equally beneficial as the in-hospital sessions in long COVID patients [51,55]. Importantly, despite the hybrid nature of the PR programme which included sessions at home following the outpatient sessions, the benefits observed were comparable with other studies where purely outpatient PR programmes were applied. It is likely that the initial outpatient programme in our cohort was of sufficient frequency and intensity, resulting in improved physiological outcomes and enabling patients to carry out the exercises at home and maintain the initial benefits.

### 4.1. Study Limitations

A few limitations should be taken into consideration while interpreting the results. The present study consisted of a relatively small sample size, and randomization was not feasible due to ethical considerations. Specifically, it was considered unethical to deny participation by patients with long COVID, and thus, the UC group was composed of long COVID patients who were unable to attend for personal reasons. Overall, patient eligibility was limited due to social and work commitments. Finally, the present study focused on the acute effects of pulmonary rehabilitation on cardiorespiratory fitness, functional capacity and quality of life. However, long-term follow-up is important in order to highlight the strengths and weaknesses of such programmes.

### 4.2. Clinical Implications

The hybrid nature of our PR programme familiarised patients with the exercise-induced symptoms during the course of the first four weeks of the face-to-face programme, most likely enhancing their confidence and future adherence to the home-based PR sessions. The promising benefits of the present hybrid PR programme may foster the implementation of other similar hybrid or fully remote PR programmes aiming to support patients with long COVID. Nevertheless, RCTs including larger and more homogenous samples and interventions should be performed in order to validate our results and lead to standardisation of PR protocols in patients with long COVID.

## 5. Conclusions

In conclusion, implementing a hybrid PR programme—comprising 8 outpatient sessions followed by 24 home-based sessions—proved beneficial for long COVID patients, leading to significant cardiorespiratory adaptations, enhanced peripheral muscle strength, improved functional capacity and a better quality of life.

## Figures and Tables

**Table 1:** Demographic characteristics of patients.

	Pulmonary Rehabilitation (PR) Group (n = 27)	Usual Care Group (n = 15)
Sex (M/F)	15/12	4/11
Age (years)	53.6 ± 12.9	60.1 ± 9.2
Height (cm)	168 ± 11	164 ± 9
Mass (kg)	81.9 ± 16.4	75.2 ± 15.3
BMI	28.9 ± 5.0	27.6 ± 4.4
Length of stay (days)	10 ± 2	10 ± 3
COVID-19 vaccine prior referral (%)	41	40
Time since hospital discharge	134 ± 69	145 ± 101
Comorbidities		
Dyslipidaemia	5	3
Hypertension	4	2
Coronary artery disease	2	1
Diabetes mellitus	3	1
Asthma	2	0
Anxiety—Depression	3	1
FEV_1_ (%predicted)	96 ± 19	90 ± 25
FEV_1_/FVC	84 ± 7	79 ± 14
TLC (litres)	5.44 ± 1.66	5.12 ± 1.81
TLC (% predicted)	95 ± 36	97 ± 30
DLco (mL/min/mmHg)	18.44 ± 7.57	16.92 ± 5.48
DLco (% predicted)	72 ± 24	71 ± 22
mMRC	2.1 ± 1.3	2.2 ± 1.2
FACIT score	27 ± 10	25 ± 12

FEV_1_: forced expiratory volume at the 1st second; FVC: forced vital capacity; TLC: total lung capacity; DLco: diffusion capacity for carbon monoxide; mMRC: modified Medical Research Council Dyspnoea Scale; FACIT: Functional Assessment of Chronic Illness Therapy.

**Table 2:** Data from CPET about peak exercise at baseline and at 12-week follow-up.

	PR Group		Usual Care Group		p-ANOVA
	Pre	Post	Pre	Post	
WR (Watt)	94 ± 46 #	113 ± 50 #*	68 ± 20	71 ± 24	0.000
WR (% predicted)	70 ± 26	86 ± 29 *	75 ± 29	78 ± 30	0.000
VO_2_ (mL/kg/min)	16.5 ± 6.2	18.9 ± 6.9 #*	13.6 ± 4.1	13.2 ± 4.4	0.006
VO_2_ (%predicted)	66 ± 17	75 ± 18 #*	65 ± 20	62 ± 19	0.007
VCO_2_ (mL/min)	1518 ± 609 #	1801 ± 736 #*	1071 ± 302	1026 ± 387	0.001
VE/VO_2_	38.4 ± 9.8	38.5 ± 7.9	35.8 ± 9.0	33.1 ± 5.5	0.342
VE/VCO_2_	34.5 ± 8.2	33.8 ± 5.7	32.9 ± 5.0	31.2 ± 2.8	0.633
RER	1.11 ± 0.07	1.15 ± 0.10	1.08 ± 0.14	1.06 ± 0.11	0.058
ΔIC (litres)	0.31 ± 0.27	0.07 ± 0.44	0.03 ± 0.29	0.02 ± 0.32	0.104
VE (litres/min)	56.7 ± 21.7 #*	65.7 ± 27.9 #	40.1 ± 13.7	36.4 ± 13.4	0.015
Vt (litres)	1.63 ± 0.61 #*	1.89 ± 0.73 #	1.26 ± 0.36	1.25 ± 0.51	0.023
Breathing frequency	37 ± 12	36 ± 8	32 ± 10	31 ± 9	0.843
Ti (sec)	0.84 ± 0.26	0.84 ± 0.20	0.92 ± 0.30	0.95 ± 0.34	0.676
Te (sec)	0.96 ± 0.32	0.92 ± 0.23	1.09 ± 0.36	1.17 ± 0.35	0.164
Ttot (sec)	1.79 ± 0.57	1.76 ± 0.41	2.01 ± 0.59	2.12 ± 0.63	0.315
Breathing reserve (%)	40 ± 19	35 ± 16	43 ± 23	49 ± 24	0.110
HR (beats/min)	127 ± 19 *	136 ± 25 #	116 ± 14	110 ± 18	0.010
HR (%predicted)	76 ± 9 *	82 ± 14 #	73 ± 9	69 ± 12	0.018
HRR	40 ± 15 *	31 ± 20 #	44 ± 16	50 ± 19	0.007
SpO_2_ (%)	97 ± 4	97 ± 3	95 ± 7	96 ± 5	0.949
O_2_pulse (mL/beat)	10.9 ± 4.1	11.6 ± 3.2	8.7 ± 2.3	8.7 ± 2.6	0.463
O_2_pulse (%predicted)	89 ± 22	95 ± 18	88 ± 21	90 ± 22	0.668
SBP (mmHg)	162 ± 30	163 ± 22	156 ± 23	163 ± 26	0.637
DBP (mmHg)	80 ± 15	84 ± 13	87 ± 12	95 ± 17	0.441
Dyspnoea	3.4 ± 2.4	2.5 ± 1.8	3.6 ± 1.2	3.0 ± 1.1	0.699
Fatigue	5.8 ± 2.3	4.7 ± 1.8	4.7 ± 2.2	4.5 ± 1.8	0.291

WR: work rate; VO_2_: oxygen uptake; VCO_2_: carbon dioxide production; VE: minute ventilation; RER: respiratory exchange ratio; ΔIC: change in inspiratory capacity (rest-peak exercise); Vt: tidal volume; Ti: inspiratory time; Te: expiratory time; Ttot: duty cycle time; HR: heart rate; HRR: heart rate reserve; SpO_2_: oxygen saturation. * denotes a statistically significant difference within the same group. # denotes a statistically significant difference between PR and usual care groups at the same time point. p-ANOVA indicates time × group differences.

**Table 3:** Cardiopulmonary exercise test data at iso-work at baseline and at 12-week follow-up.

	PR Group		Usual Care Group	
	Peak Pre	Iso-Work	Peak Pre	Iso-Work
WR (Watt)	94 ± 46		67 ± 21	
VO_2_ (mL/kg/min)	16.5 ± 6.2	15.8 ± 6.1	13.6 ± 4.1	13.4 ± 4.0
VCO_2_ (mL/min)	1475 ± 638	1380 ± 642 *	1072 ± 302	995 ± 300
RER	1.10 ± 0.09	1.06 ± 0.09 *	1.08 ± 0.14	1.03 ± 0.10 *
VE (litres/min)	55.2 ± 22.8	48.4 ± 21.7 *	40.4 ± 13.6	35.0 ± 10.2
Vt (litres)	1.587 ± 0.631	1.640 ± 0.789	1.266 ± 0.360	1.263 ± 0.476
Breathing frequency	37 ± 12	31 ± 8 *	33 ± 10	29 ± 7
Ti (sec)	0.84 ± 0.26	0.95 ± 0.26 *	0.92 ± 0.30	0.99 ± 0.34
Te (sec)	0.97 ± 0.33	1.12 ± 0.30 *	1.09 ± 0.36	1.19 ± 0.33
Ttot (sec)	1.81 ± 0.57	2.07 ± 0.53 *	2.01 ± 0.59	2.18 ± 0.60
HR (beats/min)	127 ± 18	123 ± 22	116 ± 15	108 ± 15 *
SpO_2_ (%)	97 ± 4	98 ± 2	96 ± 7	97 ± 5
O_2_pulse (mL/beat)	10.7 ± 4.2	10.5 ± 2.8	8.7 ± 2.3	9.0 ± 2.2
Dyspnoea	3.4 ± 2.4	1.9 ± 1.7 *	3.5 ± 1.0	2.8 ± 1.4
Fatigue	5.8 ± 2.3	3.4 ± 1.6 *	4.5 ± 2.1	4.4 ± 2.1

WR: work rate; VO_2_: oxygen uptake; VCO_2_: carbon dioxide production; RER: respiratory exchange ratio; VE: minute ventilation; Vt: tidal volume; Ti: inspiratory time; Te: expiratory time; Ttot: duty cycle time; HR: heart rate; SpO_2_: oxygen saturation. * denotes a statistically significant difference within the same group.

**Table 4:** Quality of life and daily physical activity levels at baseline and at 12 weeks post PR.

	PR Group		Usual Care Group		p-ANOVA
	PRE	POST	PRE	POST	
FACIT score	27 ± 10	42 ± 8 *#	25 ± 12	30 ± 13	0.002
CAT score	14 ± 8	7 ± 6 *#	17 ± 6	14 ± 7	0.044
mMRC scale	2.1 ± 1.3	0.7 ± 0.8 *#	2.2 ± 1.2	1.5 ± 1.0 *	0.046
HADS anxiety	5 ± 4	3 ± 3	9 ± 6	7 ± 5	0.927
HADS depression	6 ± 4	4 ± 4	9 ± 4	8 ± 4	0.315
EQ-5D VAS	67 ± 15	81 ± 14	60 ± 16	66 ± 18	0.101
Beck score	12 ± 9	6 ± 6	18 ± 11	15 ± 11	0.124
IES-R (PTSD) score	3.47 ± 2.43	1.86 ± 1.69	5.03 ± 2.97	3.83 ± 2.33	0.622
Steps/day	5057 ± 3223	6357 ± 3641	4554 ± 3013	4992 ± 3565	0.109

FACIT: Functional Assessment of Chronic Illness Therapy; CAT: COPD Assessment Tool; mMRC: modified Medical Research Council Dyspnoea Scale; IES-R: Impact Event Scale–Revised; PTSD: post-traumatic stress disorder. * denotes a statistically significant difference within the same group. # denotes a statistically significant difference between the PR and usual care groups at the same time point. p-ANOVA indicates time × group differences.

## Data Availability

The raw data supporting the conclusions of this article will be made available by the authors on request.

## Abbreviations

The following abbreviations are used in this manuscript:

6MWT	Six Minute Walk Test
CPET	Cardiopulmonary Exercise Test
HR	Heart Rate
PR	Pulmonary Rehabilitation
QF	Quadriceps Force
SpO_2_	Oxygen Saturation
SPPB	Short Physical Performance Battery test
UC	Usual Care
VO_2_	Oxygen Consumption
WR	Work Rate
